# Host–multiparasite interactions in amphibians: a review

**DOI:** 10.1186/s13071-021-04796-1

**Published:** 2021-06-03

**Authors:** Dávid Herczeg, János Ujszegi, Andrea Kásler, Dóra Holly, Attila Hettyey

**Affiliations:** 1 Lendület Evolutionary Ecology Research Group, Plant Protection Institute, Centre for Agricultural Research, Eötvös Loránd Research Network, Herman Ottó út 15, Budapest, 1022 Hungary; 2 Department of Systematic Zoology and Ecology, Eötvös Loránd University, Pázmány Péter sétány 1/C, Budapest, 1117 Hungary; 3 Department of Ecology, Institute for Biology, University of Veterinary Medicine, Rottenbiller utca 50, Budapest, 1077 Hungary

**Keywords:** Co-infection, *Ranavirus*, *Aeromonas hydrophila*, *Batrachochytrium dendrobatidis*, *B. salamandrivorans*, *Ribeiroia*, Helminth, Disease dynamics, Environmental factors

## Abstract

Parasites, including viruses, bacteria, fungi, protists, helminths, and arthropods, are ubiquitous in the animal kingdom. Consequently, hosts are frequently infected with more than one parasite species simultaneously. The assessment of such co-infections is of fundamental importance for disease ecology, but relevant studies involving non-domesticated animals have remained scarce. Many amphibians are in decline, and they generally have a highly diverse parasitic fauna. Here we review the literature reporting on field surveys, veterinary case studies, and laboratory experiments on co-infections in amphibians, and we summarize what is known about within-host interactions among parasites, which environmental and intrinsic factors influence the outcomes of these interactions, and what effects co-infections have on hosts. The available literature is piecemeal, and patterns are highly diverse, so that identifying general trends that would fit most host–multiparasite systems in amphibians is difficult. Several examples of additive, antagonistic, neutral, and synergistic effects among different parasites are known, but whether members of some higher taxa usually outcompete and override the effects of others remains unclear. The arrival order of different parasites and the time lag between exposures appear in many cases to fundamentally shape competition and disease progression. The first parasite to arrive can gain a marked reproductive advantage or induce cross-reaction immunity, but by disrupting the skin and associated defences (i.e., skin secretions, skin microbiome) and by immunosuppression, it can also pave the way for subsequent infections. Although there are exceptions, detrimental effects to the host are generally aggravated with increasing numbers of co-infecting parasite species. Finally, because amphibians are ectothermic animals, temperature appears to be the most critical environmental factor that affects co-infections, partly via its influence on amphibian immune function, partly due to its direct effect on the survival and growth of parasites. Besides their importance for our understanding of ecological patterns and processes, detailed knowledge about co-infections is also crucial for the design and implementation of effective wildlife disease management, so that studies concentrating on the identified gaps in our understanding represent rewarding research avenues.

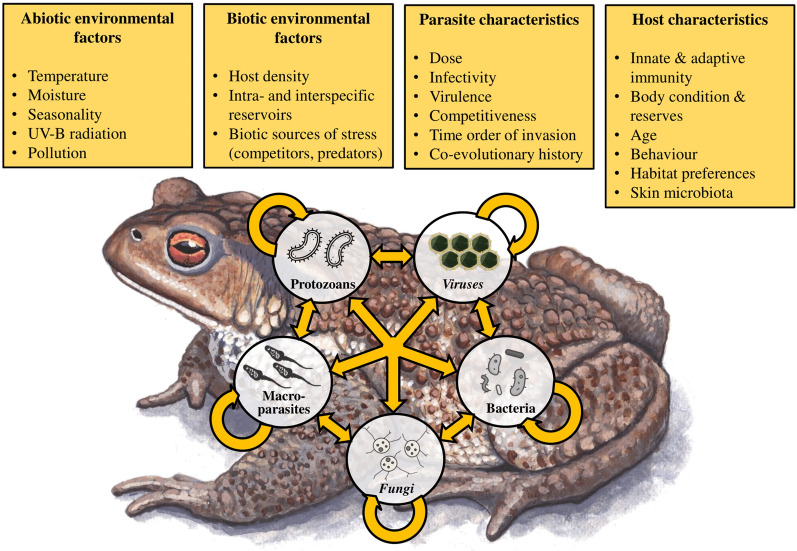

## Background

Amphibians host a wide array of microparasites (e.g., viruses, bacteria, and fungi), protists (flagellata, amoebae, sporozoans, and ciliates), and macroparasites (e.g., helminths, arthropods, and leeches) [1], many of which can have devastating effects on distinct populations and even on entire species [2, 3]. Accordingly, parasites and their interactions with amphibians have been in the focus of conservation-oriented research, but most studies have investigated one host–one parasite systems, and only a handful have considered the interactive effects resulting from the simultaneous presence of different parasites within hosts. By definition, co-infections are simultaneous infections with at least two different genotypes of parasitic organisms [4]. In this review, we consistently use the term ‘parasite’ to refer to either pathogens or parasites for easier understanding of the context. Field studies have demonstrated that the simultaneous occurrence of parasites in amphibians is a relatively common phenomenon in natural populations, so that co-infections are increasingly recognized as important drivers of disease dynamics [5–8]. Simultaneously, the high prevalence of concurrent infections renders amphibians ideal for studying the ecological and evolutionary background and consequences of co-infection [9, 10].

Frequent simultaneous encounters with multiple parasitic organisms have the potential to result in all shades of additive, antagonistic, and synergistic effects between co-infecting parasites [11, 12]. These interactions are known to influence host fitness, where the increase in parasite richness usually leads to a decrease in host survival and fitness, depending strongly on the composition of co-infecting parasites [13]. From the perspective of hosts, the presence of co-infecting parasites can cause disease synergisms via enhanced virulence [14], even if the interactions among parasites are antagonistic [15]. In general, enhanced virulence is connected with the severity of disease symptoms [15, 16]. Virulent parasites generate more symptoms, which can result in increased transmission success between hosts [17]. During co-infection, the increased parasite transmission success, i.e., a growing number of infective stages released to the environment, can alter disease dynamics [18, 19] and the epidemiology of each parasite species within the host population [18]. There is also increasing evidence for the priority effect theory, in that parasite arrival order and timing (the simultaneous and/or sequential invasion of the host) strongly influence host–multiparasite interactions [20–23].

Furthermore, considering the scale (individual, population, or community level) when evaluating co-infection outcomes is also of fundamental importance. Correlations observed on one scale (e.g., infection intensity determined on the individual level) may not be observable or may even be reversed on another scale, such as average infection intensity between sites [8, 24]. On the other hand, patterns observed on the individual level (e.g., parasite associations) are, in some cases, consistent between sites, even in taxonomically distinct clades of amphibian hosts [8]. This suggests an underlying spatial or temporal correlation of factors determining co-infections. Thus, correlated infections are not limited to particular host species and may similarly affect many amphibian communities.

Co-infecting parasites have the potential to interact with each other in direct or indirect ways. Parasites may suffer from interference competition for physical space, which can be limiting, especially in macroparasites [25, 26], or interact indirectly via resource competition [27, 28]. Moreover, parasites may suffer from the presence of other parasites via cross-reaction immunity or benefit from co-infection thanks to immunosuppression of the host [29, 30]. Finally, the population of parasites may go extinct if the host dies from the disease caused by the co-infecting agent prematurely before sufficiently matured infectious propagules can be released into the environment. Therefore, the presence of other parasites can be beneficial or detrimental for parasites during co-infection [27]. Besides, co-infection with various microparasite taxa can pave the way for horizontal gene transfer between involved parasites, resulting in new, more virulent variants [31].

Amphibians exhibit highly developed innate and adaptive immune systems that resemble those of higher vertebrates [32–34]. The innate immune system consists of granulocytes, monocytes, macrophages, dendritic cells, and natural killer cells. Its main functions are to absorb antigens and present them to B and T cells of the adaptive immune system. The complement system (humoral part of the innate immune system) aids the penetration of prokaryote and fungal cell membranes and leads the chemotaxis of phagocytes [32, 35]. Skin-secreted defensive chemicals such as antimicrobial peptides, steroids, alkaloids, or biogenic amines [36–40] provide the first line of defence against invading parasites [41, 42]. Mutualist skin bacteria (e.g., *Janthinobacterium lividum*, *Lysobacter gummosus*) can prevent infections or propagation of diseases and are also considered part of innate defence mechanisms [43–49]. Because amphibians are ectothermic animals, the effectiveness of their immune system strongly depends on environmental temperature [50–53]. Nonetheless, intrinsic factors, such as the major histocompatibility complex class II (MHCII) haplotype carried by the host, can play important roles in determining the susceptibility of amphibians to parasites [54–56]. Nonetheless, while their immune system provides effective defences against many parasites under most conditions, infectious diseases can have devastating effects on amphibian populations, especially when animals become exposed to new parasites [57, 58], and when these act in concert with other stress factors such as excessive population densities, inbreeding, habitat degradation, pollution, climate change, or other parasites [53, 59–62].

**Fig. 1:**
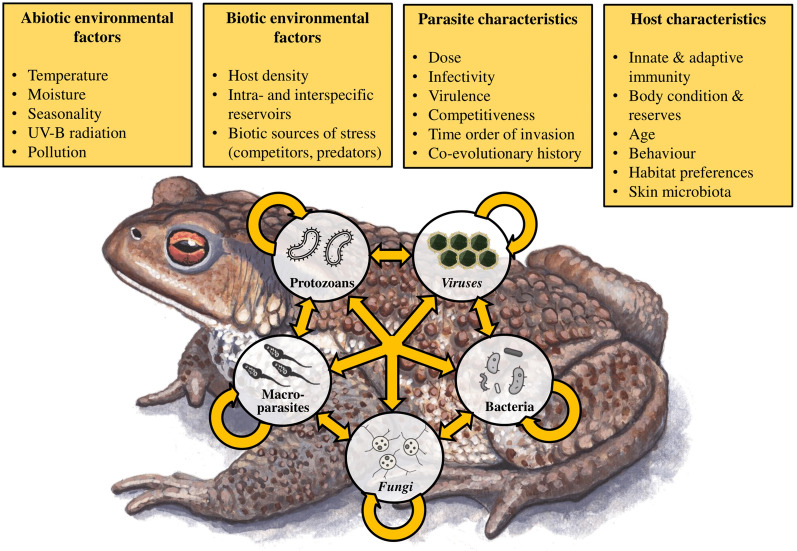
The most important factors shaping co-infections in amphibians and interactions among host and various parasites

In the present paper, we review the literature reporting on co-infection in wild and captive amphibian populations and manipulative experimental studies. We focus on parasite–parasite interactions during co-infections and how their outcomes influence disease progression in hosts. Moreover, because host responses to infection and disease must be considered within the context of the biotic and abiotic environments, we summarize what is known about environmental and intrinsic factors that may influence the virulence of co-infecting parasites (Fig. 1). We first summarize what is known about co-infection by homologous parasites (i.e., parasites belonging to phylogenetically closely related taxonomic groups). This is followed by a section on investigations on co-infections by heterologous parasites. Third, we review studies on multiple co-infections (i.e., the simultaneous presence of more than two parasite taxa). Finally, we point out major gaps in our knowledge regarding co-infections in amphibians and suggest research approaches and directions that are likely to prove fruitful in the future.

## Co-infection by homologous parasites

The co-occurrence of phylogenetically related parasites in individual hosts is common in wild populations, but relevant studies are almost exclusively descriptive field surveys (for compilations see Table 1). The small number of experimental investigations have reported both additive and antagonistic effects between parasites. Findings are context- and taxon-specific, so that there are only a few general trends observable. Some experiments found strong priority effects, especially so in the case of homologous macroparasite co-infections. Moreover, simultaneous exposure to viral or fungal parasites tends to cause higher mortality than a sequential encounter with the same agents or single infections. However, the species composition of parasites and, interestingly, the degree of relatedness between them may significantly influence interactions and, ultimately, disease outcomes. Direct competition for suitable hosts may affect fungal parasites with different virulence, selecting against less virulent variants on the population level. Also, in the case of macroparasitic organisms, direct competition for space can be the key mechanism determining outcomes besides the time order of infections.

**Table 1:** A list of studies detecting co-infections by homologous parasites in amphibians

Host	Life stage		Parasites		Consequences for hosts	Association between parasites	References
Field studies							
*Ambystoma tigrinum*	Juvenile and adult	*Acinetobacter calcoaceticus* or *A. haemolyticus*		Mass mortality		Not specified	[209]
		*Pseudomonas aeruginosa*					
*Rana muscosa*	Adult	*Aeromonas hydrophila*		Mass mortality and local extinction, bacteria isolated from a dead individual collected in the field		Not specified	[210]
		*Enterobacter aerogenes*					
*Rana muscosa*	Adult	*Enterobacter aerogenes*		Mass mortality and local extinction, bacteria isolated from a dead individual collected in the field		Not specified	[210]
		*Enterobacter agglomerans*					
*Notophthalmus viridescens*	Not specified	*Achlya* sp.		Not specified		Not specified	[211]
		*Saprolegnia parasitica*					
*Atelopus mittermeieri*	Adult	*Bd*		Isolated from a dead individual collected in the field		Not specified	[212]
		*Saprolegnia* sp*.*					
*Pseudacris regilla*	Larval	*Ribeiroia ondatrae*		Not specified		Parasites significantly more likely to co-occur in the field than expected by chance	[131]
		*Echinostoma trivolvis*					
Studies on captive populations							
*Andrias davidianus*	Egg and embryo	*Aeromonas hydrophila*		Abnormal embryonic development		Not specified	[213]
		*Cetobacterium somerae*					
		*Hafnia alvei*					
*Bombina orientalis*	Juvenile	*Aeromonas hydrophila*		Severe ocular and inner ear disease		Not specified	[214]
		*Citrobacter freundii*					
		Unknown Gram-negative bacillus					
*Bombina orientalis*	Juvenile	*Aeromonas hydrophila*		Severe ocular and inner ear disease		Not specified	[214]
		*Providencia alcalifaciens*					
		*Klebsiella oxytoca*					
*Bombina orientalis*	Juvenile	*Providencia alcalifaciens*		Severe ocular and inner ear disease		Not specified	[214]
		Unknown Gram-negative bacillus					

*Bd*: *Batrachochytrium dendrobatidis*

### Viruses

Several viruses are known to infect amphibians, but only ranaviruses (family: *Iridoviridae*) have been studied in relation to co-infection. Ranaviruses are globally emerging parasites that pose a considerable disease risk to ectothermic vertebrates and contribute to global population declines of amphibians [63–66]. What adds to the danger posed by ranaviruses is that these can infect individuals of several lower vertebrate classes, and inter-class transmissions, such as between fishes and amphibians, can occur [67, 68]. Nevertheless, ranaviruses can also be widespread without causing obvious disease or mortality [69–72]. Based on their systematic relationships, ranaviruses have been divided into two groups, the grouper-like ranaviruses (GIV-like RV) and the amphibian-like ranaviruses (ALRV) [67]. ALRVs can be further divided into three subgroups: *Ambystoma tigrinum* virus-like (ATV-like), common midwife toad virus-like (CMTV-like), and Frog virus3-like (FV3-like) viruses [73]. The most extensively studied member of ALRVs is the Frog virus-3 (FV-3) which is often highly virulent for wild and cultured amphibians [74, 75].

In an experimental study, Mihaljevic et al. [76] simultaneously exposed *Rana aurora* larvae and *Pseudacris triseriata* to ATV, FV-3, and FV-3-like virus (*Rana catesbeiana* virus; RCV-Z2) and found synergistic effects of co-infection. When larvae of *R. aurora* were co-exposed to ATV and either FV-3 or FV-3-like viruses, *Ranavirus* prevalence was higher than in groups exposed to a single virus (i.e., ATV, FV-3, or FV-3-like alone). However, co-exposure to FV-3 and FV-3-like virus did not result in a similar synergistic effect on viral infectivity, which suggests that viral identity or the degree of relatedness among co-infecting viruses may influence the spread of different viruses and ultimately shape disease outcomes during co-infection.

Extrinsic and intrinsic factors may influence the infectivity and virulence of viruses and thereby affect the co-occurrence of different viral agents. Experimental work investigating the effects of abiotic factors on ranaviruses have focused on single virus strain infections [77–79]. For example, the environmental temperature can strongly affect the replication of ranaviruses and is a key determinant of disease dynamics in wild amphibians [79]. In vitro FV-3 has been shown to replicate successfully between 8 and 30 °C, with a lower replication rate below 15 °C and the highest rate at 30 °C [80]. Replication of FV-3 ceases at 32 °C in vitro, while some virus-specific macromolecular synthesis still occurs at higher temperatures [81]. In accord with the thermophilic nature of ranaviruses, deaths caused by ranavirosis are more frequent during the warm summer months in natural populations [81]. Also, the doses of infection, acute high concentrations of stress hormones, or the developmental stage of hosts are important intrinsic factors that can significantly affect the disease outcome [82, 83]. Finally, the presence of intra- and interspecific reservoirs of the virus can also be significant for *Ranavirus* transmission [84, 85]. For instance, adult and juvenile *Ambystoma tigrinum* can serve as reservoirs for ATV transmission by repeatedly introducing the virus into the larval population, thereby maintaining the infection in populations between years, even if during dry periods breeding ponds desiccate and virus transmission would otherwise be disrupted [84]. Nonetheless, how extrinsic and intrinsic factors influence co-infections with parasitic viruses has so far remained unexplored.

### Bacteria

Disease-causing bacteria can be obligate or opportunistic parasites of amphibians, capable of inducing localized and systemic infections [1, 86, 87]. Bacterial infections reported in wild and captive amphibians are mainly induced by Gram-negative bacteria [1, 86]. Historically, some amphibian die-offs were attributed to infections with multiple bacteria without adequate examination of the primary agent responsible for the mortality event and without testing for other parasites [1, 74, 88]. Further ambiguities stem from the fact that samples were mostly isolated from dead hosts. The identified microbes could be at least partly saprophytic bacteria that rapidly invade and spread throughout carcasses [1, 86]. Although the course of infections with single bacterial taxa has been studied in detail [1, 89], relatively few surveys followed concurrent bacterial infections in amphibians, and almost all were descriptive investigations performed in the field (Table 1), not allowing for conclusions on within-host interactions between bacterial parasites or on their role in disease progression.

To our knowledge, the only experimental study on co-infection with bacteria reported great variation in the number and composition of microbe species in individuals of *Rana catesbeiana* [90]. Carr et al. [90] did not perform experimental infections but merely manipulated environmental temperature and assessed bacterial taxa present in diseased animals. Sick animals showed classical signs of septicaemia and were pithed after developing symptoms. Individuals that became diseased at 24 or 17 °C had 10–25 different bacteria in their guts and blood, whereas individuals that became diseased at between 3 and 12 °C had only 3–5 different bacteria. The list of bacteria observed in members of the ‘cold’ group contained mostly Gram-negative species, such as *Aeromonas hydrophila*, *Pseudomonas*, or *Acinetobacter* spp., which are considered pathogenic for frogs. While these were also present in the ‘warm’ group, they formed a minority beside many other bacterial taxa. Carr et al. [90] concluded that hibernation-like conditions may be advantageous for potentially parasitic bacteria by lowering numbers of competitors.

As suggested by Carr et al. [90], the temperature is perhaps the most critical factor affecting bacterial co-infections in amphibians, which may be partly due to its effect on the amphibian immune system as well as on the survival and growth rate of bacteria. It has been shown that varying temperatures can enhance the probability of bacterial septicaemia [88]. Also, the combination of low temperature and reduced food supplies favours colonization by cryophilic, potentially parasitic bacteria [90]. In addition to the effect of temperature, endogenous biological rhythms of the neuroendocrine system may also influence the immune system of amphibians, resulting in seasonal variation in the bacterial assemblage inhabiting them [51]. Furthermore, environmental stress can lead to immunosuppression, resulting in enhanced probability of infection by opportunistic bacterial parasites [86]. Nonetheless, while the nature and outcomes of co-infections involving bacteria will remain notoriously difficult to assess and forecast because of the extreme diversity, very high reproduction rate, and omnipresence of bacteria, studying the course of (co-)infections involving the most virulent taxa, determining the major factors that influence these processes, and uncovering the bacterial agents that contribute to mass die-offs will certainly provide highly valuable insights for disease ecology and conservation biology.

### Fungi

Several fungal parasites can cause severe problems in amphibian populations and are likely related to co-infections [91]: Mucormycosis is caused by *Mucor amphibiorum* and is typically a subclinical systemic disease, but the consequently weakened condition of the host can facilitate the emergence of other parasites [92], resulting in co-infection. The common gut commensal *Basidiobolus ranarum* can invade its amphibian host percutaneously, causing the fatal disease called basidiobolomycosis [93], and may also facilitate secondary bacterial septicaemia [92, 94]. Further, various pigmented filamentous fungi (e.g., *Cladosporium*, *Phialophora*, *Exophiala*, and *Fonsecaea*) can cause chromomycosis [95], where the infection is thought to occur opportunistically [1], and these fungi may also act as secondary invaders. Finally, *Saprolegnia* and other water moulds (Oomycetes) are responsible for the disease saprolegniasis, which is often deadly by itself and has been linked to amphibian declines [96], but can also be accompanied by primary or secondary bacterial infections [1, 92]. However, most research on fungal parasites of amphibians has focused on *Batrachochytrium dendrobatidis* (*Bd*) and the more recently discovered *B. salamandrivorans* (*Bsal*), the causative agents of the disease chytridiomycosis which has led to massive die-offs globally [97, 98].

Recently, the concurrent presence of both chytrids was documented in metamorphosed *Salamandra salamandra* in the Eifel Mountains, Germany, which was accompanied by a local mass-mortality event [99]. In an experimental study, Longo et al. [100] demonstrated that adult *Notophthalmus viridescens* that were simultaneously co-exposed to *Bd* and *Bsal* experienced higher mortality compared to single *Bd* or *Bsal* exposure. Mortality was intermediate when exposure to the two agents was sequential, regardless of which was added first. This suggests that the immune response mounted against the first agent provides some protection against the second. Alternatively, the two chytrid fungi may be in direct competitive interaction with each other (e.g., for limited space or via allelochemicals). Furthermore, exposure of *N. viridescens* to an exceedingly high *Bd* dose increased subsequent susceptibility of the host to *Bsal* [100]. Thus, the outcome of co-infection with the two chytrids appears to depend on the relative timing of exposures and the dose of zoospores and may result in both synergistic and antagonistic effects. These findings, along with the hazard of horizontal gene transfer potentially resulting in new, more virulent strains [31], suggest that the co-occurrence of both chytrids can pose extreme risks to wild and captive populations.

The highly virulent *Bd* Global Pandemic Lineage (GPL) has been spread mainly by human activities throughout all continents except Antarctica [101, 102], and co-infection with different lineages of *Bd* on *Flectonotus fissilis* has also been observed [103]. Experimental co-infection of adult *Hymenochirus curtipes* with two lineages of *Bd* (GPL and Brazil) demonstrated higher zoospore production by *Bd* GPL than *Bd* Brazil, where this difference further increased with disease progression. This is likely to result in competitive exclusion and the replacement of the less virulent strain on the population scale [104]. Under natural circumstances, co-infection has reportedly led to hybridization between *Bd* GPL and *Bd* Brazil [101, 102], where hybrid *Bd* was isolated from both larval and adult individuals of *Hylodes cardosoi* [105, 106]. Experimental evidence indicated that the hybrid lineage could cause higher mortality than the parental lineages in *Brachycephalus ephippium* and *Lithobates sylvaticus*. Still, the prevalence of this hybrid genotype and its effect on mortality can fall between that of parental *Bd* lineages in other frog species (i.e., *Ischnocnema parva* and *Dendropsophus minutus*). Thus, hybrid virulence appears to be context-specific and largely depends on host characteristics, such as the species of host, immune functions, habitat choice, or geographical distribution [107], but most likely also on which *Bd* lineages hybridize and on the resulting genotype of the hybrid.

Reports on co-infections between *Bd* and fungal agents other than chytrids in the field are scarce (Table 1). Experimental evidence for synergism between *Bd* and a non-chytrid fungal agent, *Achlya* sp., in larval *Hyliola regilla* was provided by Romansic and colleagues [6]. The authors suggested that the germ tubes of *Achlya* sp. may disrupt epidermal layers and thereby facilitate colonization by *Bd*. Interestingly, the synergism was abolished in the presence of a glyphosate-based herbicide, which had no significant effect on the host’s survival alone [6].

Several environmental and intrinsic factors have been demonstrated to modulate the outcome of fungal diseases in amphibians [32, 92]. Pulses of high temperature (28–30 °C) and a dry and warm season can reduce infection loads by chytrids and *Saprolegnia* species in ectothermic vertebrates [108–110]. Conversely, humid and moist conditions can favour the infectivity of *Bd* [111] and, by preventing desiccation, are likely advantageous for other fungal parasites as well. The intensity of ultraviolet B radiation correlates inversely with *Bd* prevalence in Spanish anuran populations [112], but can negatively affect the survival of *Saprolegnia*-infected eggs [113]. The sensitivity of amphibians to fungal parasites may also strongly depend on the life stage. For example, eggs are the most sensitive to *Saprolegnia* [1, 96], and tadpoles are less susceptible to *Bd* infection than metamorphosed individuals [110]. The behaviour of hosts can also influence bacterial disease outcomes: communal deposition of egg masses may enhance mortality due to *Saprolegnia* infection [114], and aggregation of hosts can facilitate parasite transmission in the case of amphibian chytrids [115, 116]. How these factors affect the outcome of co-infections by multiple fungal agents has remained unknown and would require detailed investigations.

### Protists

Information regarding members of the paraphyletic group of protists involved in co-infections in amphibians is exceptionally scarce. In the last two decades, many mass mortality events were reported in North American *Lithobates sphenocephala* tadpoles [117, 118], where the causative agent was identified to be an intracellular protist parasite of the phylum Perkinsozoa (genus *Perkinsus*) belonging to the superphylum Alveolata [119, 120]. It appears that the parasite exhibits cryptic genetic diversity and is widespread globally [120]. Infection with *Perkinsus* causes pathology in the liver of tadpoles [118], an organ which is also known to be targeted by other alveolate parasites like *Nematopsis temporariae* [121–123] or *Goussia* sp. [124, 125]. Indeed, macrophages in the liver sinusoids of *Rana dalmatina* and *Rana temporaria* collected from the Czech Republic were reportedly co-infected with *N. temporariae* oocysts and *Goussia noelleri* oocysts. However, the role amphibians play in the life cycle of the latter alveolate species and their importance in disease progression has remained unclear [122, 123]. Various other protist parasites, i.e., amoebae (e.g., *Entamoeba* spp.), ciliates (*Tetrahymena* spp.), flagellates (trypanosomes), and sporozoans (*Eimeria* and *Isospora* spp.), are capable of inducing diseases in amphibians [1]. However, how homologous co-infections involving protist parasites progress, how protists interact with one another in the host, and what extrinsic factors influence the outcomes remains entirely obscure and requires further studies.

### Macroparasites

Macroparasites are the most widely studied parasites of wild amphibians. Historically, studies on helminth parasites of amphibians (we refer here to helminths as worm-like members of the phyla Annelida, Platyhelminthes, Nematoda, and Acanthocephala) were mostly restricted to local or regional faunistic surveys or simply species descriptions. Nowadays, however, the attention has shifted away from purely descriptive studies towards more quantitative approaches [126, 127] and the investigation of processes shaping community patterns [13, 128–130]. Amphibians serve as intermediate or definitive hosts for a variety of helminth parasites in aquatic and terrestrial food webs. Therefore, helminth infracommunities (i.e., communities of parasite infrapopulations in a single host) are ideal systems for investigations of host responses to simultaneous parasitic infections and within-host interactions between co-infecting parasites.

What is known about host–multi-helminth systems mainly stems from research on a North American hylid and its trematode parasites. *Pseudacris regilla* is an intermediate host of larval stages of *Ribeiroia ondatrae* and *Echinostoma trivolvis*, and this association is commonly observed in wild populations [8, 13, 131, 132]. Although these helminths have different infection sites within the host (epithelial tissue for *Ribeiroia* and kidney for *Echinostoma*), multiscale field studies combined with manipulative experiments showed that during co-infection, these parasites negatively affected the persistence of one another, and this was likely due to cross-immunity [131]. It has also been shown that the diversity of the parasite community has a significant effect on disease dynamics in this system. Johnson and Hoverman [13] exposed *P. regilla* to six different trematode species (*R. ondatrae*, *E. trivolvis*, *Alaria* sp., *Cephalogonimus americanus*, *Clinostomum attenuatum*, and an undescribed echinostome magnacauda) and found that an experimental increase in parasite species richness decreased the overall infection success within the host, including that of the most virulent helminths (i.e.,* R. ondatrae* and *E. trivolvis*). Nonetheless, increased parasite species richness caused increased host mortality even when the numbers of the most virulent species were held constant, but pathology decreased when additional species replaced virulent ones and total helminth numbers were held constant [13]. Hoverman et al. [21] sequentially exposed *P. regilla* to *R. ondatrae* and *E. trivolvis* and found that when the two helminth species were added simultaneously, there was no competition between them; however, when they added them sequentially, infection success of the second parasite was decreased, but only when exposure to *E. trivolvis* preceded that of *R. ondatrae*. These findings support the hypothesis that the sequence of parasite encounters can significantly shape the competition between parasites, and this aligns with results obtained for other host–parasite systems [20, 100, 133, 134]. Because encounters between helminths and hosts are likely to be highly stochastic, it is difficult to predict the outcome of co-infections at the population level when priority effects are strong.

Also, arthropods such as chiggers, ticks, and blowflies are facultative or obligate ectoparasites of amphibians, taking blood, damaging cutaneous tissues, and potentially acting as vectors of microparasites [1]. A striking, usually fatal case of parasitism by an arthropod is myiasis caused by the calliphorid blowfly *Lucilia bufonivora*: females lay their eggs close to the nostrils or wounds of amphibians, and the hatching larvae feed on the tissues of the host [135]. Even in sublethal cases, the large, open wounds caused by the maggots may serve as the entrance point for secondary infections by microparasites [136]. How co-infections involving these macroparasites affect amphibian individuals and populations and how the co-infecting parasites interact within their hosts has remained virtually unknown.

The likelihood of (co)infection by macroparasites can strongly depend on environmental characteristics. The transmission of free-living stages of helminths with indirect life cycles, i.e., helminths that use intermediate host(s) during development, can be strongly influenced by biotic and abiotic environmental factors [137–139]. For instance, the thermal environment can modulate the susceptibility of *Xenopus laevis* to infection with a polystomatid monogenean (*Protopolystoma xenopodis*): significantly lower numbers of helminths survived in the urinary bladder of the host at higher (25 °C) than at lower (15 °C) experimental temperature [140]. It is worth noting that some exogenous stressors, such as toxic chemicals or metals, may have stronger negative impacts on helminth parasites than their amphibian hosts [141, 142]. For instance, certain helminths interacted with agricultural disturbance to alter the physiology and immune competence of *R. catesbeiana* [143]. Moreover, intraspecific predation between *Ambystoma macrodactylum* enhances the frequency of severe limb malformation caused by *R. ondatrae* [144]. Finally, the interactive effects of predation risk and other stressors like herbicides (e.g., atrazine) can also shape host–parasite dynamics [60]. Unfortunately, manipulative experimental studies testing the effects of these factors on co-infections with macroparasites are lacking.

## Co-infection by heterologous parasites

Hosts can also be co-infected with parasites belonging to different taxonomic groups (for a list of case studies see Table 2). Although our knowledge of this phenomenon has remained fragmentary, evidence suggests that co-infection by several parasitic taxa can be devastating for affected host populations. However, it can also have surprisingly mild consequences, so that outcomes are often difficult to predict. Similarly to co-infections caused by homologous parasites, clear patterns in the case of heterologous parasites are also scarce. Experimental studies reported strong priority effects when macroparasitic and viral parasites co-occurred in amphibian hosts. Studies performed in natural populations frequently co-detected viral and fungal parasites and documented both additive and antagonistic interactions between them. From the perspective of hosts, individuals co-infected with a viral and a fungal pathogen tend to display higher parasite loads, resulting in more severe disease symptoms than individuals infected with just one agent, and disease progression influenced by the time spans between parasite exposures. Finally, how the host’s immune system affects interactions and which type of parasite wins the race for physical space and other vital resources remains challenging to generalize because of the often asymmetric nature of interactions and stochasticity in the resulting patterns.

**Table 2:** A list of studies detecting co-infections by heterologous parasites in amphibians

Host	Life stage	Parasites	Consequences for hosts	Association between parasites	References
Field studies					
*Rana catesbeiana*	Larval	*Rv* (FV-3-like) + alveolate (species not specified)	Larval mortality	Not specified	[182]
*Lithobates sphenocephalus*					
*Anaxyrus boreas*	Larval, metamorph	*Rv* + *Echinostoma* sp.	Not specified	Negative correlation between infection intensity of *Rv* and *Echinostoma* sp.	[8]
*Rana catesbeiana*					
*Alytes obstetricans*	Larval and metamorph	*Bd* + *Rv*	Larval mortality	*Bd* and *Rv* infection intensities were not significantly correlated	[168]
*Ichthyosaura alpestris*					
*Ambystoma talpoideum*	Not specified	*Bd* + *Rv*	Not specified	Not specified	[61]
*Rana catesbeiana*	Larval and adult				
*Lithobates sphenocephalus*	Adult				
*Anaxyrus terrestris*	Adult	*Bd* + *Rv*	Not specified	*Bd* prevalence was higher in individuals that were positive for *Rv*	[61]
*Craugastor fitzingeri*	Metamorph	*Bd* + *Rv*	Not specified	A positive association between *Bd* and *Rv* prevalence	[165]
*Cryptobranchus alleganiensis*	Not specified	*Bd* + *Rv*	Tail lesions, fused toes, and healed abrasions on the body of co-infected animals	Not specified	[153]
*Eurycea bislineata*	Adult	*Bd* + *Rv*	Not specified	No association between *Bd* and *Rv* prevalence	[159]
*Rana catesbeiana*					
*Lithobates clamitans*					
*Hyla versicolor, H. chrysoscelis*	Juvenile	*Bd* + *Rv*	Bloating, swelling, discolouration, lacerations, and morphological abnormalities	Not specified	[160]
*Lithobates sylvaticus*					
*Pseudacris maculata, P. triseriata*					
*Lissotriton boscai*	Adult	*Bd* + *Rv*	Not specified	Not specified	[154]
*Hypsiboas gladiator*	Adult	*Bd* + *Rv*	Not specified	*Rv* infection intensity was marginally associated with the probability of *Bd* infection	[164]
*Rhinella manu*					
*Pristimantis* spp*.*					
*Rana catesbeiana*	Larval and adult	*Bd* + *Rv*	Not specified	No association	[61]
*Lithobates sphenocephalus*					
*Lithobates sylvaticus*	Larval, juvenile and adult	*Bd* + *Rv*	No visible signs of *Bd* and *Rv* infections were observed	Not specified	[155]
*Notophthalmus viridescens*	Adult	*Bd* + *Rv*	An individual collected alive showed small red tail lesions, another individual found dead in the field	Not specified	[163]
*Lithobates clamitans*	Larval	*Bd* + *Rv*	Not specified	Not specified	[163]
*Pelophylax caralitanus*	Not specified	*Bd* + *Rv*	Not specified	Not specified	[215]
*Pseudacris regilla*	Larval, metamorph	*Bd* + *Rv*	Not specified	A positive correlation between *Bd* and *Rv* infection intensity	[8]
*Telmatobius marmoratus*	Adult	*Bd* + *Rv*	Not specified	No association	[164]
*Rana catesbeiana*	Adult	*Bd* + *Rv*	Not specified	Higher *Bd* infection intensity in co-infected individuals	[166]
*Lithobates sphenocephalus*					
*Alytes obstetricans*	Metamorph	*Bd* + *Rv* (CMTV-like)	Isolated from a dead individual collected in the field	Not specified	[154]
*Bufo spinosus*	Metamorph	*Bd* + *Rv* (CMTV-like)	Isolated from a dead individual collected in the field	Not specified	[154]
*Hyla molleri*	Adult	*Bd* + *Rv* (CMTV-like)	Not specified	Not specified	[154]
*Xenopus laevis*	Adult	*Bd* + *Rv* (FV-3)	Euthanized after live capture	Not specified	[157]
*Bufo bufo*	Metamorph	*Bd* + *Rv* (FV-3-like)	Not specified	Not specified	[156]
*Rana muscosa*	Larval	*Bd* + *Rv* (FV-3-like)	Mass mortality in larval population associated with both parasites	Not specified	[158]
*Boana pulchella*	Adult	*Bd* + *Amphibiocystidium* sp.	Not specified	Not specified	[216]
*Eleutherodactylus cooki*	Adult	*Bd* + *Carios* sp.	Male-biased co-infections due to male parental care: nesting sites are highly infested by ticks, and also suitable for *Bd* persistence	Not specified	[100]
*Boana pulchella*	Adult	*Bd* + *Valentines rwandae*	Seasonal variability in prevalence, with distinct peaks for the two parasites	No association	[217]
*Lissotriton helveticus*	Adult	*Bd* + *Rv* + *Dermocystidium* sp.	Isolated from a dead individual collected in the field	Not specified	[218]
*Rana aurora*	Larval	*Bd* + ciliates + flagellates + *Ribeiroia* sp. + other macroparasites	*Bd*-infected tadpoles had significantly lower parasite species richness than those not infected	Diverse macroparasite fauna negatively influenced *Bd* prevalence	[198]
*Pseudacris regilla*	Larval, metamorph	*Bd* + *Alaria* sp.	Not specified	Infection intensities negatively correlated between *Bd* and *Alaria* sp.	[24]
*Pseudacris regilla*	Larval, metamorph	*Bd* + *Echinostoma* sp.	Not specified	Infection intensities negatively correlated between *Bd* and *Echinostoma* sp.	[24]
*Rana catesbeiana*	Larval, metamorph	*Bd* + *Echinostoma* sp.	Not specified	Infection intensities negatively correlated between *Bd* and *Echinostoma* sp.	[8]
*Pseudacris regilla*					
*Anaxyrus boreas*	Larval, metamorph	*Ribeiroia ondatrae* + *Echinostoma* sp.	Not specified	A positive correlation between infection intensity of macroparasites	[8]
*Taricha torosa*					
*Pseudacris regilla*					
Studies on captive populations					
*Rana catesbeiana*	Larval, juvenile	*Rv* + Myxosporidium (most likely *Sphaerospora* sp.)	*R. catesbeiana* tadpole showed symptoms like oedema and erythema	Not specified	[69]
*Lithobates clamitans*					
*Rana catesbeiana*	Larval	*Bd* + *Myxidium* sp.	Not specified	Not specified	[219]
*Dendrobates auratus*	Adult	*Bd* + *Rv* + *Aeromonas hydrophila*	Gross changes e.g., sloughing skin, proliferative ulcerations, and swollen and pale liver, were observed in dead animals	Not specified	[161]
*Phyllobates terribilis*					
*Pyxicephalus adspersus*					
*Rhacophorus dennysi*					

*Bd*: *Batrachochytrium dendrobatidis*; CMTV-like: common midwife toad virus-like virus; FV-3: Frog virus-3 virus; FV-3-like: Frog virus 3-like virus; *Rv*: *Ranavirus*

### Viruses and bacteria

In the late 1980s, mass mortality events were reported in *Rana temporaria* populations in the UK, where infected animals suffered from skin lesions and ulcerations as well as from systemic haemorrhages [145]. Some investigated *R. temporaria* individuals were simultaneously infected by an iridovirus-like particle (*Ranavirus*) and *A. hydrophila* [145]. The described syndromes with lesions could be consistent with the red-leg disease symptoms, which primarily attributed the cause of death to bacterial septicaemia, putatively caused by *A. hydrophila* [146]. However, a few studies [145, 147] suggested that *Ranavirus* mainly causes the disease and that the source (tissue homogenate vs cultured virus from naturally diseased frogs) of viral agents and the method of exposure were the factors that primarily determined disease outcomes [147]. The question has remained unanswered whether the red-leg disease and associated mortalities are caused by the primary viral infection or the additional presence of secondary invaders, such as the bacterial agent. Although the bacterial infections are likely common in *Rv*-infected amphibians [145, 148], we know of no reports on co-infections of amphibians with other systems.

### Viruses and fungi

*Batrachochytrium dendrobatidis* and ranaviruses, the most devastating parasites of amphibians [64, 97, 149, 150], can co-occur [7, 151–153] and cause repeated severe mass die-offs [154]. Although several studies have reported the co-infection of amphibians with *Bd* and *Ranavirus* under natural conditions [155–160] and captive populations [161–164], little information exists on within-host interactions between these agents.

Field studies suggested that co-occurring *Bd* and *Ranavirus* can, in some cases, have positive effects on each other [8, 61, 165, 166]. Still, this interaction was not confirmed in other cases [61, 159, 164, 167] or even turned negative [165, 168] (Table 2). A lower *Bd* or *Ranavirus* infection intensity in individuals co-infected by both parasites compared to single infections may arise from an enhanced immune response of the host or the interspecific competition between the agents [166]. On the other hand, higher infection intensity can be explained by immunosuppression in hosts; but how simultaneous viral and fungal infection alters the host immune response has remained theoretical due to lack of experimental data. In a recent experiment, Ramsay and Rohr [169] demonstrated that the length of the time spans between exposures to *Ranavirus* and *Bd* can have a decisive influence on disease progression: post-metamorphic *Osteopilus septentrionalis* previously infected with *Bd* exhibited increased viral loads relative to hosts exposed only to *Ranavirus*, and this effect increased with time since exposure to *Bd*. Furthermore, *Bd* and *Rv* co-infection risk can be influenced by the developmental stage and the reproductive behaviour of the hosts: the aquatic life stage of frogs is more exposed to these agents than terrestrial life stages [84, 170]. Nonetheless, Love et al. [61] observed a higher prevalence of *Bd* and *Ranavirus* in terrestrial individuals of *L. sphenocephalus* than in aquatic larvae, and co-infection was detected in terrestrial *Pristimantis* spp. as well [164]. Finally, elevated physiological stress periods, such as during breeding or when animals pass through sensitive developmental windows, may make amphibians temporally more vulnerable to co-infection [160, 164].

Habitat characteristics may also influence the susceptibility of amphibians to *Bd* and *Ranavirus*. In southern Peru at altitudes between 900 and 2400 m a.s.l., Warne et al. [164] observed a rapid decline in *Bd* prevalence in adult *Pristimantis* spp. above 2100 m, while the prevalence of *Ranavirus* was the lowest below 1200 m, and co-infected adults were only present at elevations ranging between 1200 and 2100 m. Wetland pollution (e.g., ammonia) can be responsible for increased odds of the concurrent occurrence of *Bd* and *Ranavirus*, perhaps by overwhelming the immune system of amphibians [160]. Additionally, in adults of *A. terrestris*, Love et al. [61] detected higher odds of co-infection with *Bd* and *Ranavirus* in metal-contaminated wetlands than reference wetlands. Furthermore, intensive agricultural land use (i.e., cattle accessing wetlands) can also lead to increased *Ranavirus* prevalence [70] and, hence, co-infections involving *Ranavirus*. Seasonality may also influence the probability of co-infection by affecting the prevalence of both *Bd* and *Ranavirus*: the prevalence of *Bd* is usually the highest during cool and moderately warm months [159, 165]; in contrast, *Ranavirus* peaks were present in the warmest period(s) of the year [171, 172]. Talbott and colleagues [160] found that *Bd* and *Ranavirus* co-infection probability was significantly higher during spring than summer or fall. On the other hand, Olori et al. [159] observed large inter-annual variation in prevalence, but they did not detect significant differences across the months of each year. These results suggest that spatiotemporal factors jointly influence the incidence of co-infections with *Bd* and *Ranavirus* and their distribution. Nonetheless, controlled experimental studies scrutinizing the interactions between these two parasites during pathogenesis and the environmental factors that influence the outcomes would be necessary for the establishment of cause-and-effect relationships. Also, all studies have so far focused on the joint occurrence of *Bd* and *Ranavirus*. To the best of our knowledge, co-infections involving other fungal and viral parasites have not yet been reported in amphibians.

### Bacteria and fungi

The rather limited literature on co-infections involving parasitic bacteria and fungi has so far not delivered unambiguous evidence for interactive relationships between these microparasites. This is surprising, because in environments other than amphibian hosts, members of these two taxa are often fierce competitors, while they can also be symbionts [173]. In relation to amphibians specifically, some studies suggest that bacteria may often be the secondary invaders that follow fungal infections [1, 87], so that co-infections may occur frequently. Reed et al. [174] observed *Chlamydia pneumoniae* infection along with *Bd* in a breeding colony of *Xenopus tropicalis*, where more than 90 % of the animals died. Finally, Rivas [175] found that *Bd*-infected adults of *Lithobates yavapaiensis* and *Pseudacris ornata* were frequently co-infected with *A. hydrophila*, but the bacterial infection was only detectable on the skin of dead individuals, which suggests an opportunistic invasion of carcasses post-mortem.

Experimental studies suggest that some skin bacteria of amphibians can prevent infection with *Bd* [44, 176, 177], but sometimes *Bd* can inhibit the growth of antifungal bacteria [178]. This suggests that there may be an evolutionary arms race between fungi and bacteria colonizing amphibian skin. In an experimental study, Taylor et al. [94] exposed *Bufo hemiophrys* to the fungus *Basidiobolus ranarum* causing mycotic dermatitis. The primary infection was soon accompanied by secondary infection with *A. hydrophila*, *Pseudomonas* spp., and other bacteria, and became fatal in most cases when *B. ranarum* infection occurred via injuries caused experimentally to adult hosts.

The environment can significantly impact interactions between hosts, members of their associated microbiome, and invading bacterial and fungal parasites. The microclimate of the immediate environment, pollution, pH, CaCO_3,_ and conductivity can influence these complex interactions as reviewed in Bernardo-Cravo et al. [179]. Nonetheless, it is important to note that both bacterial and fungal agents can form biofilms, making them highly resistant to antimicrobials and environmental factors [180, 181].

### Heterologous co-infections involving parasitic protists

Information on heterologous co-infections involving parasitic protists is even rarer than similar information on homologous co-infections. There is growing evidence that emerging alveolate infections that are affecting an increasing number of amphibian populations worldwide [120, 123] can cause high mortality rates, especially when they are accompanied by viral outbreaks [182]. However, the exact drivers of the die-offs and the interaction between these parasites have so far remained unclear. Co-infection involving fungi and protists were already documented at the beginning of the twentieth century, when De Beauchamp [183] found small zoospore-producing sporangia, potentially of a *Batrachochytrium* sp., along with a parasite similar to *Dermocystidium pusula*, a unicellular eukaryotic parasite (formerly assumed to be a fungus), on *Lissotriton* (formerly *Triturus*) *helveticus.* A century later, co-infections with *Bd* and other dermocystid parasites were confirmed from Uruguay in North American fish hatcheries (Table 2). Finally, we do not know any studies reporting on co-infections involving parasitic protists and bacteria or macroparasites in amphibians.

### Viruses and macroparasites

Macroparasite infestations are likely to be accompanied by infections with several types of viruses in wild populations, but documented cases of such co-infections [7] and relevant experimental studies so far have all involved ranaviruses [169, 184]. Nonetheless, viruses (and more generally, microparasites) causing subclinical symptoms are frequently overlooked, and their prevalence is likely to be underestimated [185], while research on interactions between viruses and macroparasites under natural conditions is entirely lacking.

A laboratory experiment examined the effects of arrival time and order of a larval echinostome (*Echinoparyphium* sp.) and ranaviruses (FV-3) on the survival of *Hyla versicolor* tadpoles and the interspecies interactions between the parasites [184]. Interactions among parasites were asymmetric: when *H. versicolor* was exposed first to *Echinoparyphium* sp. and subsequently to FV-3, infection intensity of FV-3 decreased, while after exposure in reverse order such an effect on *Echinoparyphium* sp. infection intensity was not apparent. Furthermore, the sequence of exposure affected the survival of hosts: if exposure to *Echinoparyphium* sp. preceded that to FV-3 by 10 days, hosts enjoyed elevated survival compared to hosts infected solely with FV-3. In a mesocosm experiment involving larvae of four amphibian species, Wuerthner et al. [184] observed the same priority effect in three out of four hosts that were first exposed to *Echinoparyphium* sp. and subsequently to FV-3: FV-3 loads were decreased by 19, 27, and 28% in *H. versicolor*, *Lithobates pipiens*, and *Pseudacris crucifer*, respectively. These findings support the hypothesis that macroparasite infection can sometimes reduce the replication rate of microparasites in amphibians. More often than not, however, macroparasite infection is likely to facilitate subsequent invasion by viruses, just as reported for higher vertebrates [11, 186, 187] and for macroparasite-bacterium co-infections [86]. Indeed, Ramsey and Rohr [169] found that FV-3 infection load was increased if *Osteopilus septentrionalis* was previously infected with the macroparasite *Aplectana hamatospicula* compared to hosts infected with FV-3 alone. However, what extrinsic and intrinsic factors determine the outcome of co-infections involving viruses and macroparasites beyond priority effects remains in most cases to be evaluated.

### Bacteria and macroparasites

Bacterial and macroparasitic co-infections in amphibians are frequent and can cause severe pathologies. For example, infections by monogeneans, nematodes, or acanthocephalans can cause damage to the host’s outer and inner integument and thereby pave the way for secondary infections by bacteria [86]. Also, leeches and pentastomid crustaceans that feed on the blood of amphibians can reportedly transmit parasitic bacteria [86]. Nonetheless, to the best of our knowledge, investigations focussing on the interplay between bacterial infections and macroparasite infestation are entirely lacking.

It is worth noting that several reports exist on hyperparasitism, when a parasite, in this case a macroparasite, is parasitized by another bacterial, parasitic agent. A handful of surveys have investigated *Rickettsia* species that infect ticks associated with different species of amphibians [188–190]. Cotes-Perdomo et al. [189] assessed the bacterial infection status of tick-infested amphibian hosts and stressed that none of the host tissue samples analysed was positive for *Rickettsia*. However, other studies provided evidence that *Rickettsia* species can infect amphibians [191–193]. It has remained an open question whether these bacteria spread horizontally or vertically between ticks and whether they can cause disease in tick- and *Rickettsia*-infested amphibians.

### Fungi and macroparasites

Chiggers (*Hannemania* sp. and *Eutrombicula alfreddugesi*) and *Bd* were reported from the same population of *Tlalocohyla smithii*, but evidence for a confirmed case of co-infection was not delivered [194]. Therefore, the authors concluded that chiggers may not facilitate *Bd* infection because *Bd* prefers cooler and moist conditions, unlike these arthropods. However, co-infection by *Bd* and ticks was confirmed in Puerto Rico in another case study (Table 2).

In an experimental study, presumably accidental co-infection with *Bd* and a monogenean ectoparasite, *Gyrodactylus jennyae*, was documented in *R. catesbeiana* tadpoles [195]. Although *Bd* infection was not confirmed except for one individual, experimental exposure to *Bd* resulted in enhanced risk of mortality due to *G. jennyae* infection. This synergism may have resulted from immunosuppression or stress caused by the presence of *Bd* [195]. Furthermore, in a recent experiment, post-metamorphic *Osteopilus septentrionalis* showed higher nematode (*A. hamatospicula*) infection intensity after *Bd* exposure compared to single *A. hamatospicula* infection [169]. When the order of infections was reversed, *Bd* load was lower in hosts infected with *A. hamatospicula*, and *Bd* load correlated positively with the time span between exposure events [169]. In contrast, such an interaction between *Bd* infection and infestation by the trematode *R. ondatrae* was not confirmed experimentally in either larval or post-metamorphic *P. regilla* [12]. Thus, while *Bd* may frequently occur jointly with macroparasites, and co-infection was indeed documented by several studies, detailed experimental investigations on the factors determining disease outcomes in the case of co-infection with fungi and macroparasites, as well as information on co-infections between macroparasites and parasitic fungi other than *Bd*, are lacking.

## Multiple co-infections

Many parasites can parasitize multiple host species, and almost all hosts can be co-infected with multiple parasites. This is not a recent discovery; a series of case studies have confirmed this general rule over the last decades. However, the diversity of parasites that can infect a single amphibian host, the different ecological characteristics of parasites, the direct and indirect competition between parasites, and the varying intensity and effectiveness of host responses and their parasite-specific susceptibilities make co-infections involving multiple parasites highly complex. Consequently, the outcomes of multiple co-infections are extremely difficult to predict reliably, and this is exacerbated by the paucity of relevant experimental studies. The only general trend that can be drawn from the available data is that infection success can depend on the competitiveness of the parasitic agents, with arrival order and timing of the invasion also playing decisive roles.

Veterinary diagnoses can provide highly valuable snapshots about the co-occurrence of parasites in captive and wild amphibians, which may trigger more detailed experimental investigations [69]. For instance, Miller et al. [161] observed the joint occurrence of *Aeromonas hydrophila*, *Bd*, and *Ranavirus* in four species of amphibians in a captive breeding facility. Moribund individuals showed the following symptoms: lethargy, loss of appetite, gross lesions, sloughed skin, and rarely dermal ulcerations. Also, Hill et al. [196] detected *A. hydrophila*, *Bd*, *Mycobacterium* spp., and *Contracaecum* spp. in a *Xenopus laevis* female originating from the wild in Santiago, Chile, and held in captivity in the United States. The co-infected individual showed abnormal skin shedding (dysecdysis), stupor, and cutaneous ulcerations throughout the body. Similar reports on multiple infections in the field are scarce (Table 2).

The only experimental investigation on multiple infections we know of focussed on the outcomes of competition among parasites: Romansic et al. [12] experimentally infected *Pseudacris regilla* metamorphs with *Bd*, *Ribeiroia* sp., and *Achlya flagellate* and observed only weak interactive effects between parasites, where synergism was only documented between *Bd* and *Ribeiroia* sp. All treatment combinations with the parasites induced deformities. The treatment group involving all agents had the highest proportion of deformed individuals (77 %), displaying deformities like cephalic and axial oedema, missing or extra toes, and missing or extra hind limbs [12]. Beyond the competitiveness of involved parasites, however, their arrival order [184] and the timing of exposure, i.e., in which life stage the host encounters which parasite [21], are also likely to influence virulence and the interactions between co-infecting agents. Further experimental studies scrutinizing within-host interactions among more than two types of parasites are lacking and would provide novel and highly valuable insights into the ecology of multiple co-infections.

## Conclusions

Understanding how co-infections drive pathologies remains a fundamental knowledge gap in wildlife disease ecology. Because the medical condition and fitness of the host depend on parasite establishment, persistence, and replication, research addressing the relative importance and interrelations of the factors driving these characteristics, and thereby the outcome of within-host parasite interactions, is needed. The species-specific infectivity and virulence of parasites, host condition, environmental characteristics, competitiveness of parasites within hosts, immunosuppression, cross-reaction immunity, and costs of mounting an immune response paid by hosts are all known to shape the outcome of co-infections [197], but detailed knowledge about these factors and processes regarding amphibians is exceptionally scarce. In the present review we identified the main groups of parasites that are at least partly responsible for population declines or for inducing disease in amphibians. We also report that the outcomes of co-infections often appear inconsistent, and general trends are as yet difficult to identify.

Field surveys assessing infections with various parasites usually aim to determine the cumulative number of parasitic species present in a habitat or host population. Still, the individual level of infections is typically not discussed, making it difficult to draw conclusions on the presence of actual co-infections, community-level interactions between parasites, and effects on host–parasite dynamics [7, 198]. Also, the assessment of among-parasite interactions at the level of host communities can be misleading, because statistical relationships can be suggestive of interactions that do not manifest at the level of definitive hosts. For example, the prevalence of parasites transmitted together horizontally or vertically will be positively associated at the community level even if they do not benefit from each other’s presence in their definitive host [199]. In other words, the presence of particular parasites may correlate positively within or between host individuals, even if the interactions between them are antagonistic [13, 24]. In other cases, a frequent co-occurrence of certain parasites may be caused by one parasite facilitating the establishment of the other or by a background factor that provides a beneficial environment for both parasites. Moreover, if co-occurring parasites are highly virulent, and effects on the host are additive or synergistic, (co-)transmission of parasites may be prevented by the premature death of the host, and co-infections may remain undetected because of their brief duration. Apparent interactions among co-infecting parasites can also arise via altered host behaviour: infections that affect activity or avoidance behaviours can influence defences against other parasites, e.g., behavioural fever [200–202], and can affect encounter rates with additional parasites [203, 204]. Also, chance events such as the time-order in which hosts encounter different parasites can decisively influence the outcome of co-infections, partly because the success of the secondary parasite depends on whether the primary parasite stimulates or inhibits the host’s immune response, and partly because time-order effects may overrule among-parasite differences in competitiveness [205]. Consequently, field surveys can only provide a starting point in evaluating interactions among parasite species and their species-by-species and cumulative effects on amphibian hosts, especially so in multihost–multiparasite systems. Therefore, field surveys combined with manipulative and/or multiscale experimental studies are needed to predict disease outcomes initiated by multiple parasitic organisms in natural amphibian populations.

Empirical studies may be complemented by modelling approaches. Predictive models [206, 207] are parameterized by spatial, environmental, or presence-absence data to predict the distribution and (co-)occurrence of parasites. On the other hand, multi-response models provide the opportunity to model associations and correlations directly [208], which is an especially promising approach when the aim is to investigate co-infection in multihost–multiparasite systems and to compare among-parasite associations at the host level with data obtained on the scale of populations [8]. Quantitative reviews (meta-analyses) can also be invaluable in drawing major and general conclusions and thereby furthering a research field, but these are only feasible once sufficient empirical data are available. Because of the paucity of relevant studies, performing such analyses on the results of investigations focusing on co-infections in amphibians would be premature just yet. We first need to gather more empirical data and expand the taxonomic coverage of studies. More consistent experimental protocols and reporting of standardized effect sizes would facilitate the performance of future meta-analyses. Such carefully executed investigations would provide critically important information for the parameterization of theoretical models. Ultimately, understanding within-host interactions between parasite species and the effects of co-infections on hosts may help in devising effective treatment strategies and preventing population declines and extinctions.

## Data Availability

Not applicable.

## Abbreviations

ALRV: Amphibian-like *Ranavirus*
ATV-like virus: *Ambystoma tigrinum* virus-like virus
*Bd*: *Batrachochytrium dendrobatidis*
*Bsal*: *Batrachochytrium salamandrivorans*
CMTV-like virus: Common midwife toad virus-like virus
FV-3 virus: Frog virus-3 virus
FV-3-like virus: Frog virus-3-like virus
GIV-like RV: Grouper-like *Ranavirus*
GPL: Global pandemic lineage
*Rv*: *Ranavirus*
